# Commentary: Mortality Risk of Antidiabetic Agents for Type 2 Diabetes With COVID-19: A Systematic Review and Meta-Analysis

**DOI:** 10.3389/fendo.2021.825100

**Published:** 2022-01-10

**Authors:** Li-Min Zhao, Xie-Hui Chen, Mei Qiu

**Affiliations:** ^1^Department of Geriatric Medicine, Shenzhen Longhua District Central Hospital, Shenzhen, China; ^2^Department of General Medicine, Shenzhen Longhua District Central Hospital, Shenzhen, China

**Keywords:** COVID- 19, death, type 2 diabetes mellitus, SGLT2I, glp1 agonists, DPP4 (dipeptidyl peptidase 4) inhibitors

## Introduction

In a recent article (1), Kan and colleagues assessed the association between use of four kinds of hypoglycemic agents (i.e., metformin, sulfonylurea, dipeptidyl peptidase-4 inhibitors [DPP4i], and insulin) and risk of COVID-19 mortality in patients with type 2 diabetes mellitus (T2DM), by performing meta-analysis based on eligible studies. However, the authors did not address the association of two novel classes of hypoglycemic agents (i.e., sodium-glucose cotransporter-2 inhibitors [SGLT2i] and glucagon-like peptide-1 receptor agonists [GLP1RA]) with COVID-19 mortality, due to the limited number of included studies regarding SGLT2i and GLP1RA. Nowadays, more and more studies have been published, mainly targeting the relationship between SGLT2i or GLP1RA use and COVID-19 mortality risk (2–10). Therefore, we intended to include these recent studies regarding SGLT2i and GLP1RA to conduct an updated meta-analysis to address their association with COVID-19 mortality in T2DM patients. Moreover, several new cohort studies (2–4, 8, 11–14) targeting the relationship of DPP4i use with COVID-19 death risk have been published after Kan et al.’s meta-analysis (1). Thus, in this updated meta-analysis we would also include these new DPP4i studies to reassess the association between use of DPP4i and risk of COVID-19 death in T2DM patients.

## Methods

Eligible studies for inclusion in this updated meta-analysis were cohort studies that enrolled T2DM patients with COVID-19 and estimated the COVID-19 mortality risk of use of DPP4i, SGLT2i, or GLP1RA, compared to no use of DPP4i, SGLT2i, or GLP1RA, respectively. The comparison of SGLT2i/GLP1RA use versus no SGLT2i/GLP1RA use included that of SGLT2i/GLP1RA use versus DPP4i use, whereas the comparison of DPP4i use versus no DPP4i use did not include that of DPP4i use versus SGLT2i/GLP1RA use. Outcome of interest was COVID-19 death, and we preferred to use the most long-term outcome data. For example, if 7-day death (i.e., death within 7 days following hospital admission due to COVID-19) and 30-day death were both reported in included studies, we would use 30-day death. We searched PubMed, Cochrane Library, and Embase from the inception date of databases to November 21, 2021, using the main search terms: “COVID-19”, “SARS-CoV-2”, “type 2 diabetes mellitus”, “DPP4i”, “SGLT2i”, “GLP1RA”, “death”, and “mortality”. Two authors independently performed study selection, quality assessment, and data extraction; and a third author addressed their divergences when necessary. The quality of included studies was assessed using the Newcastle-Ottawa Scale (15), according to which a quality score for included studies ranged from 0 to 9, with a score of ≥7 being regarded as “high quality”.

We extracted the estimators of relative risk (RR) and 95% confidence interval (CI) from included studies to perform meta-analysis. We preferentially extracted hazard ratio as RR, followed by risk ratio and odds ratio. Meta-analysis was conducted based on the comparisons of DPP4i versus Non-DPP4i, SGLT2i versus Non-SGLT2i, and GLP1RA versus Non-GLP1RA, respectively. Meta-analysis was done using a random-effects model when I^2^ ≥50%, or using a fixed-effects model when I^2^ <50%. We drew funnel plots and performed Egger’s test to judge publication bias. Sensitivity analysis was conducted *via* the leave-one-out method (omitting one study each time), to evaluate the robustness of meta-analysis results. All statistical analyses were completed in Stata/MP 16.0.

## Results

We finally included 18 cohort studies (2–14, 16–20) in this meta-analysis. All of them were evaluated as “high quality”. The detailed features of included studies and the outcome data extracted from them are presented in **Table S1**. Meta-analysis incorporating 16 studies (2–4, 7–14, 16–20) involving 549817 participants showed that compared to Non-DPP4i use, DPP4i use was significantly associated with a 17% reduction in COVID-19 mortality risk (RR 0.83, 95% CI 0.71-0.98, P =0.023; **Figure 1.1**). Meta-analysis incorporating 6 studies (2–7) involving 275468 participants showed that compared to Non-SGLT2i use, SGLT2i use was significantly associated with a 22% reduction in COVID-19 mortality risk (RR 0.78, 95% CI 0.61-0.98, P =0.035; **Figure 1.2**). Meta-analysis incorporating 6 studies (2, 3, 6, 8–10) involving 140859 participants showed that compared to Non-GLP1RA use, GLP1RA use was significantly associated with a 25% reduction in COVID-19 mortality risk (RR 0.75, 95% CI 0.60-0.94, P =0.013; **Figure 1.3**).

**Figure 1:**
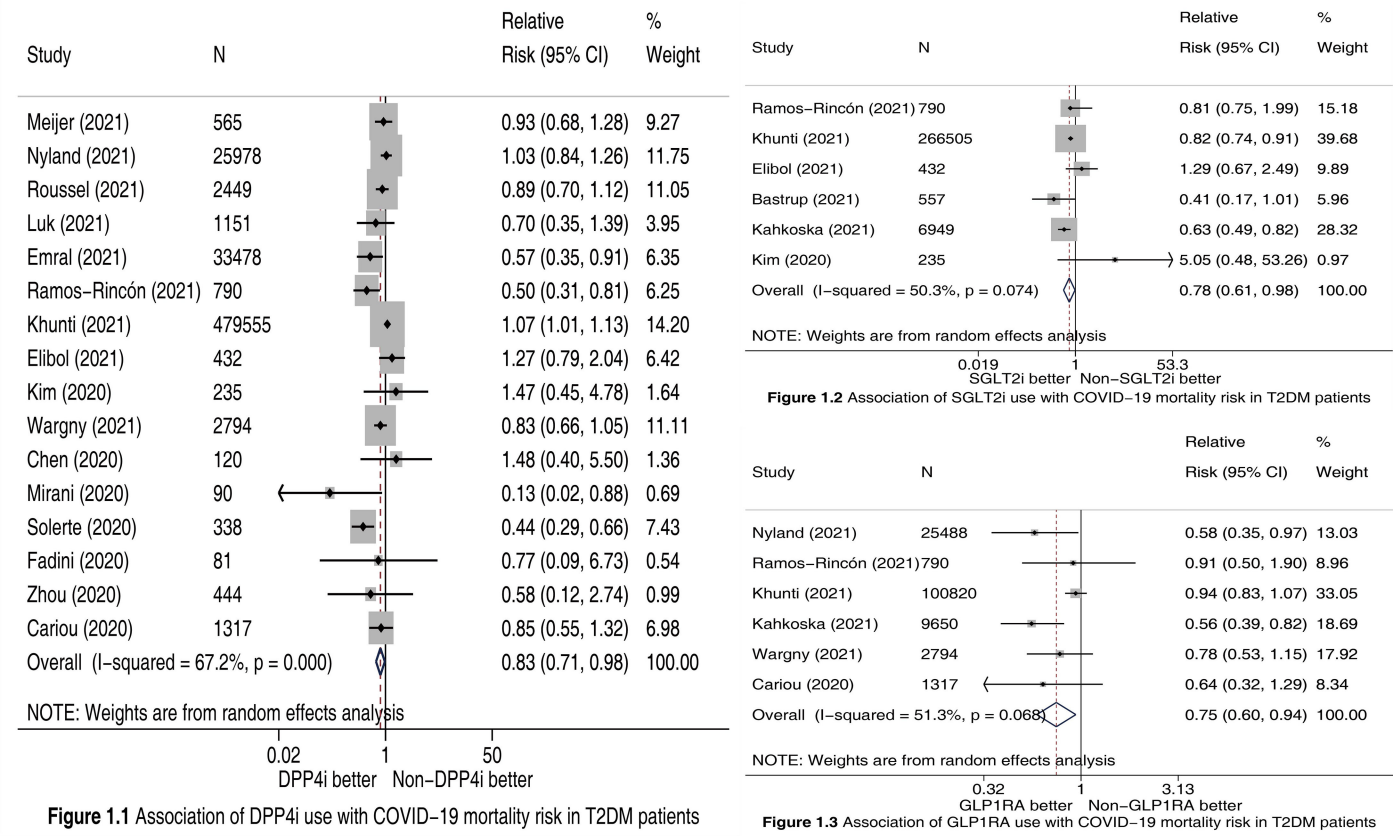
Forest plots illustrating the association of DPP4i **(Figure 1.1)**, SGLT2i **(Figure 1.2)**, and GLP1RA **(Figure 1.3)** use with COVID-19 mortality risk in T2DM patients. DPP4i, dipeptidyl peptidase-4 inhibitors; SGLT2i, sodium-glucose cotransporter-2 inhibitors; GLP1RA, glucagon-like peptide-1 receptor agonists; T2DM, type 2 diabetes mellitus; CI, confidence interval.

Publication bias was observed in the meta-analysis of DPP4i (P_Egger_ =0.012; **Figure S1**), and was not observed in the meta-analyses of SGLT2i (P_Egger_ =0.885; **Figure S2**) and GLP1RA (P_Egger_ =0.092; **Figure S3**). The results of sensitivity analysis for DPP4i (**Figure S4**), SGLT2i (**Figure S5**), and GLP1RA (**Figure S6**) were similar with primary analysis results, which suggested the robustness of meta-analysis results.

## Discussion

In Kan and colleagues’ article (1) the authors did not perform meta-analysis to assess the association between use of SGLT2i and GLP1RA and risk of COVID-19 mortality since they included only one SGLT2i study (7) and two GLP1RA studies (9, 10). On the contrary, we conducted meta-analysis respectively based on 6 SGLT2i studies (2–7) and 6 GLP1RA studies (2, 3, 6, 8–10), and accordingly revealed the significant association of SGLT2i use with a 22% reduction in COVID-19 mortality risk, and of GLP1RA use with a 25% reduction in that risk. Moreover, Kan et al.’s meta-analysis (1) did not include 8 recently-published DPP4i studies (2–4, 8, 11–14), which limited its statistical power. Accordingly, a statistically significant difference between DPP4i and Non-DPP4i was not observed in that meta-analysis (1). In contrast, this updated meta-analysis included 16 DPP4i studies (2–4, 7–14, 16–20) including the 8 recent ones (2–4, 8, 11–14), and therefore with sufficient statistical power identified that DPP4i use versus Non-DPP4i use was significantly associated with a 17% reduction in COVID-19 mortality risk.

One strength of this study is that sensitivity analyses suggested the robustness of meta-analysis results although possible publication bias was observed in DPP4i’s meta-analysis. Oppositely, this study has two main weaknesses. First, we did meta-analyses based on cohort studies. Although we extracted the adjusted RRs and 95% CIs from included studies to perform meta-analyses, there must be certain confounding factors unadjusted. Thus, relevant randomized trials are needed to verify our findings. Second, we failed to compare various hypoglycemic agents in COVID-19 death risk, and future studies comparing them are clinically meaningful.

Three new classes of hypoglycemic agents (i.e., SGLT2i, GLP1RA, and DPP4i) were significantly associated with reduced risk of COVID-19 death in T2DM patients with COVID-19, which highlights the potential of these new drugs used to prevent COVID-19 death in T2DM patients with COVID-19. Further studies are needed to assess whether there are significant differences in the risk of COVID-19 death among various kinds of hypoglycemic agents.

## Author Contributions

Design: MQ. Conduct/data collection: L-MZ, X-HC, and MQ. Analysis: L-MZ, and X-HC. Writing manuscript: L-MZ. Reviewing and editing manuscript: X-HC, and MQ. All authors contributed to the article and approved the submitted version.

## Conflict of Interest

The authors declare that the research was conducted in the absence of any commercial or financial relationships that could be construed as a potential conflict of interest.

## Publisher’s Note

All claims expressed in this article are solely those of the authors and do not necessarily represent those of their affiliated organizations, or those of the publisher, the editors and the reviewers. Any product that may be evaluated in this article, or claim that may be made by its manufacturer, is not guaranteed or endorsed by the publisher.

## Supplementary Material

Supplementary Figure 1 Test of publication bias for the meta-analysis of DPP4i versus Non-DPP4i.

Supplementary Figure 2 Test of publication bias for the meta-analysis of SGLT2i versus Non-SGLT2i.

Supplementary Figure 3 Test of publication bias for the meta-analysis of GLP1RA versus Non-GLP1RA.

Supplementary Figure 4 Sensitivity analysis of DPP4i versus Non-DPP4i.

Supplementary Figure 5 Sensitivity analysis of SGLT2i versus Non-SGLT2i.

Supplementary Figure 6 Sensitivity analysis of GLP1RA versus Non-GLP1RA.

Supplementary Table 1 Features of included studies and outcome data extracted from them.
