# Supplementary figures and images for: Commentary: Mortality Risk of Antidiabetic Agents for Type 2 Diabetes With COVID-19: A Systematic Review and Meta-Analysis
Source: Front Endocrinol (Lausanne). 2022 Jan 10;12:825100. doi: 10.3389/fendo.2021.825100 (PMC8785330; doi:10.3389/fendo.2021.825100)

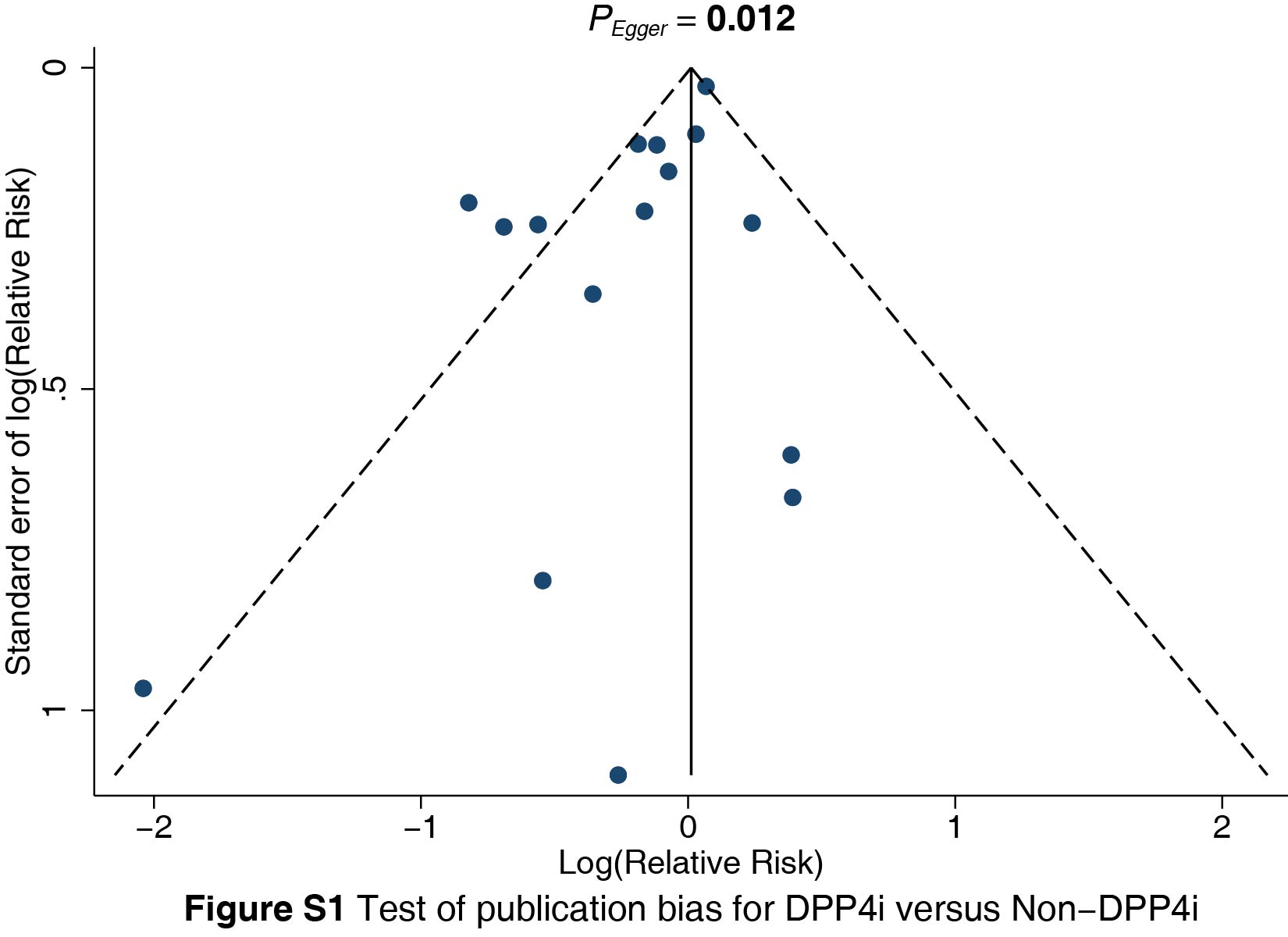

Supplement: Supplementary Figure 1 — Test of publication bias for the meta-analysis of DPP4i versus Non-DPP4i. [file Image_1.jpg]

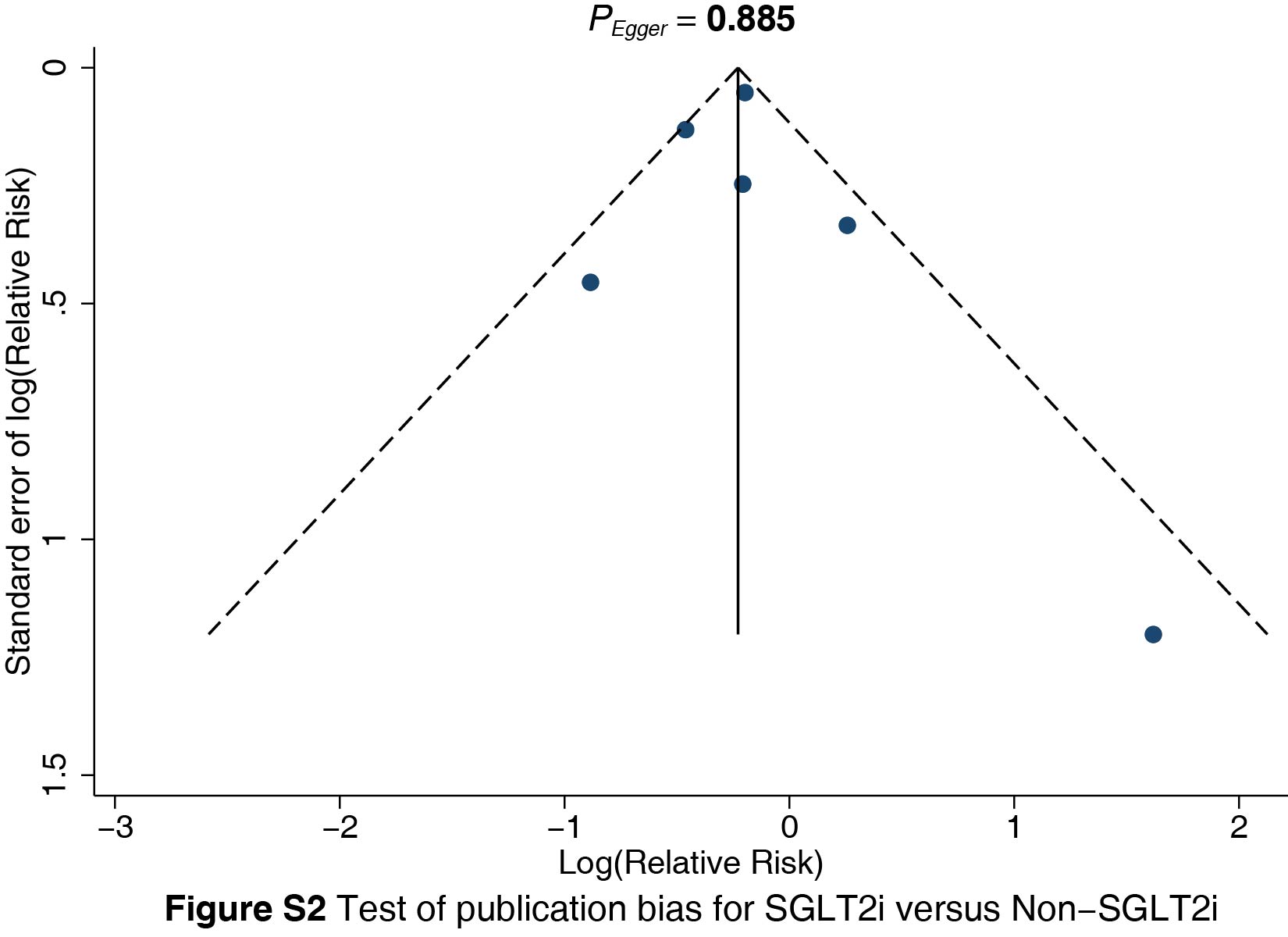

Supplement: Supplementary Figure 2 — Test of publication bias for the meta-analysis of SGLT2i versus Non-SGLT2i. [file Image_2.jpg]

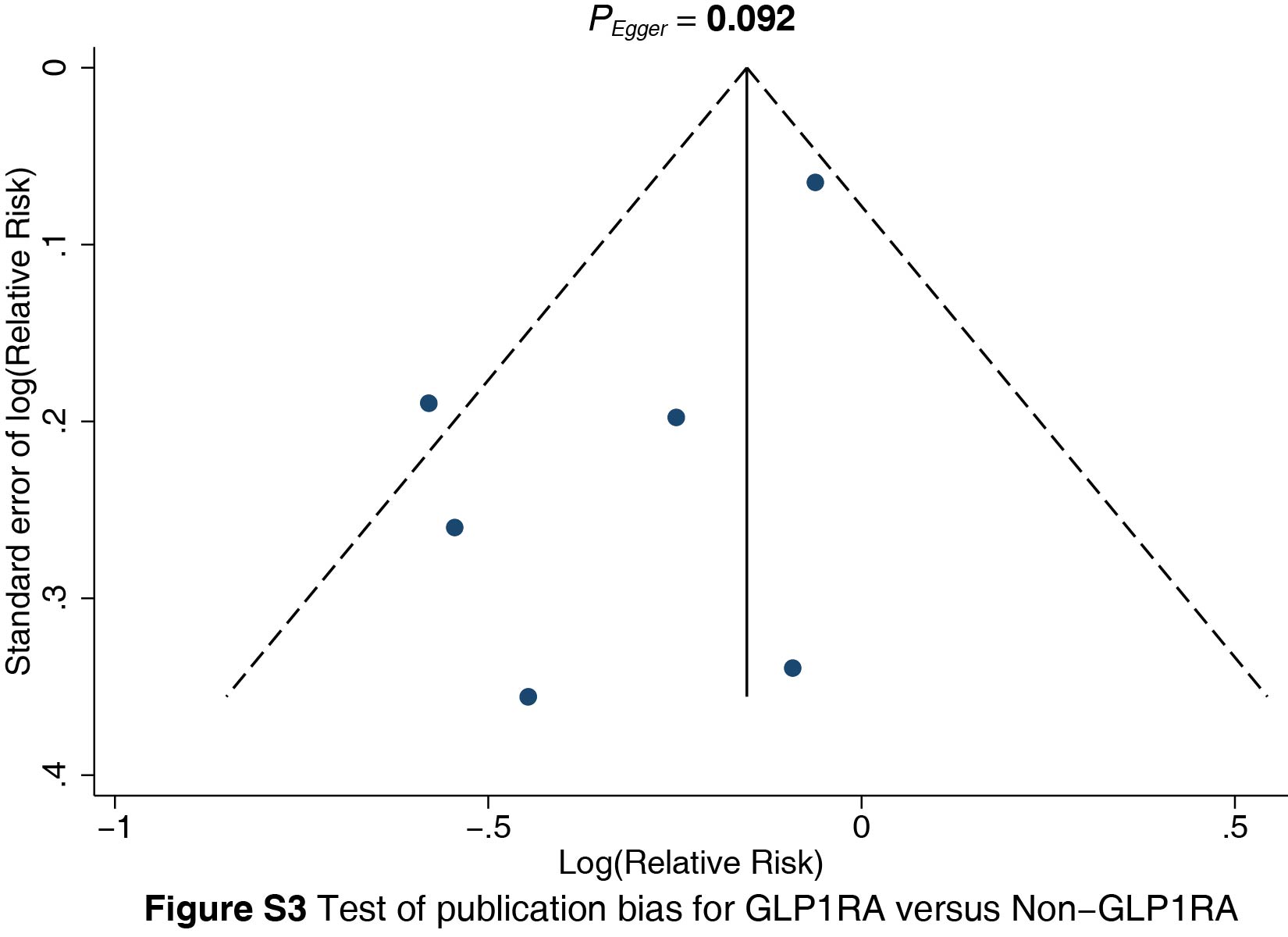

Supplement: Supplementary Figure 3 — Test of publication bias for the meta-analysis of GLP1RA versus Non-GLP1RA. [file Image_3.jpg]

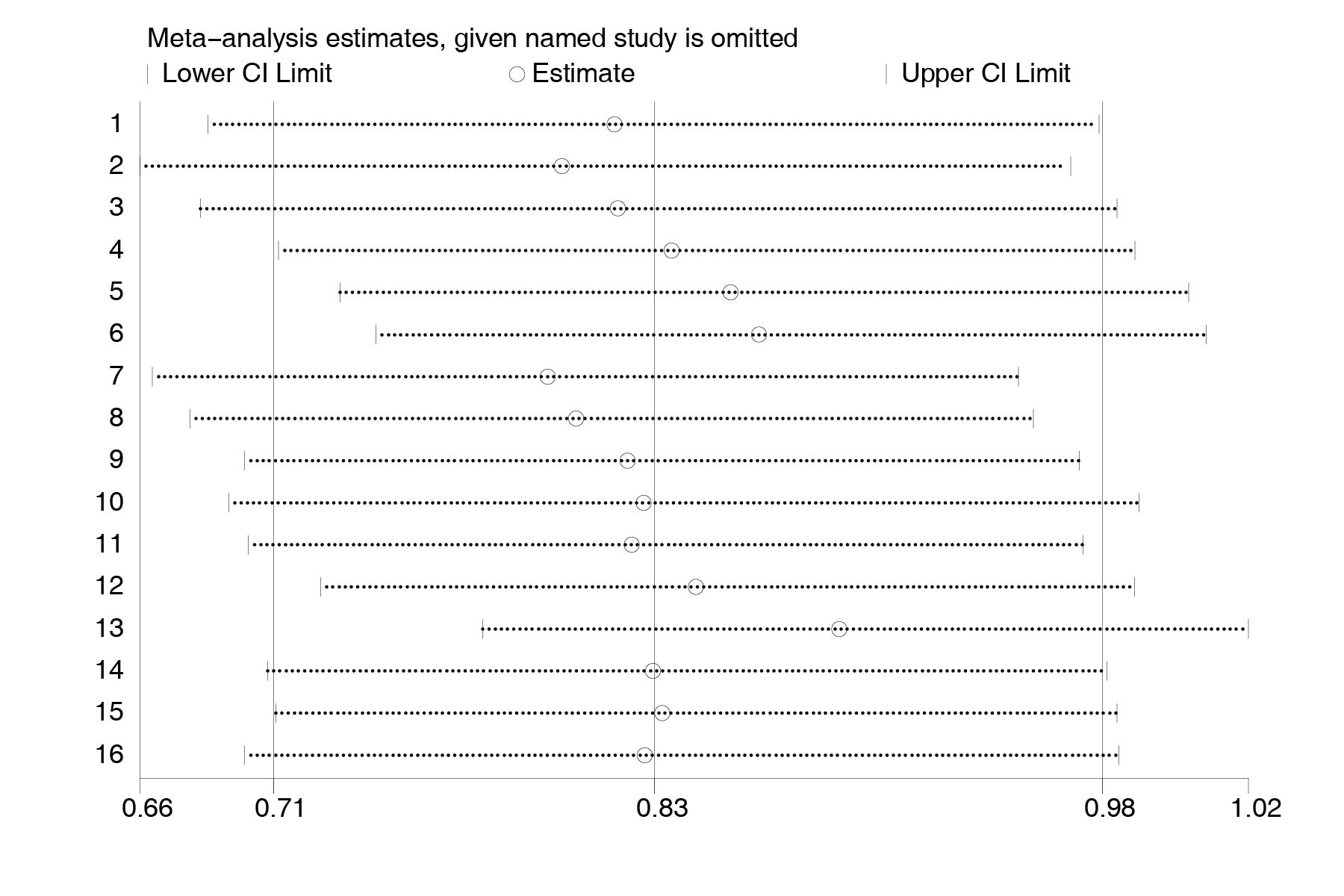

Supplement: Supplementary Figure 4 — Sensitivity analysis of DPP4i versus Non-DPP4i. [file Image_4.jpeg]

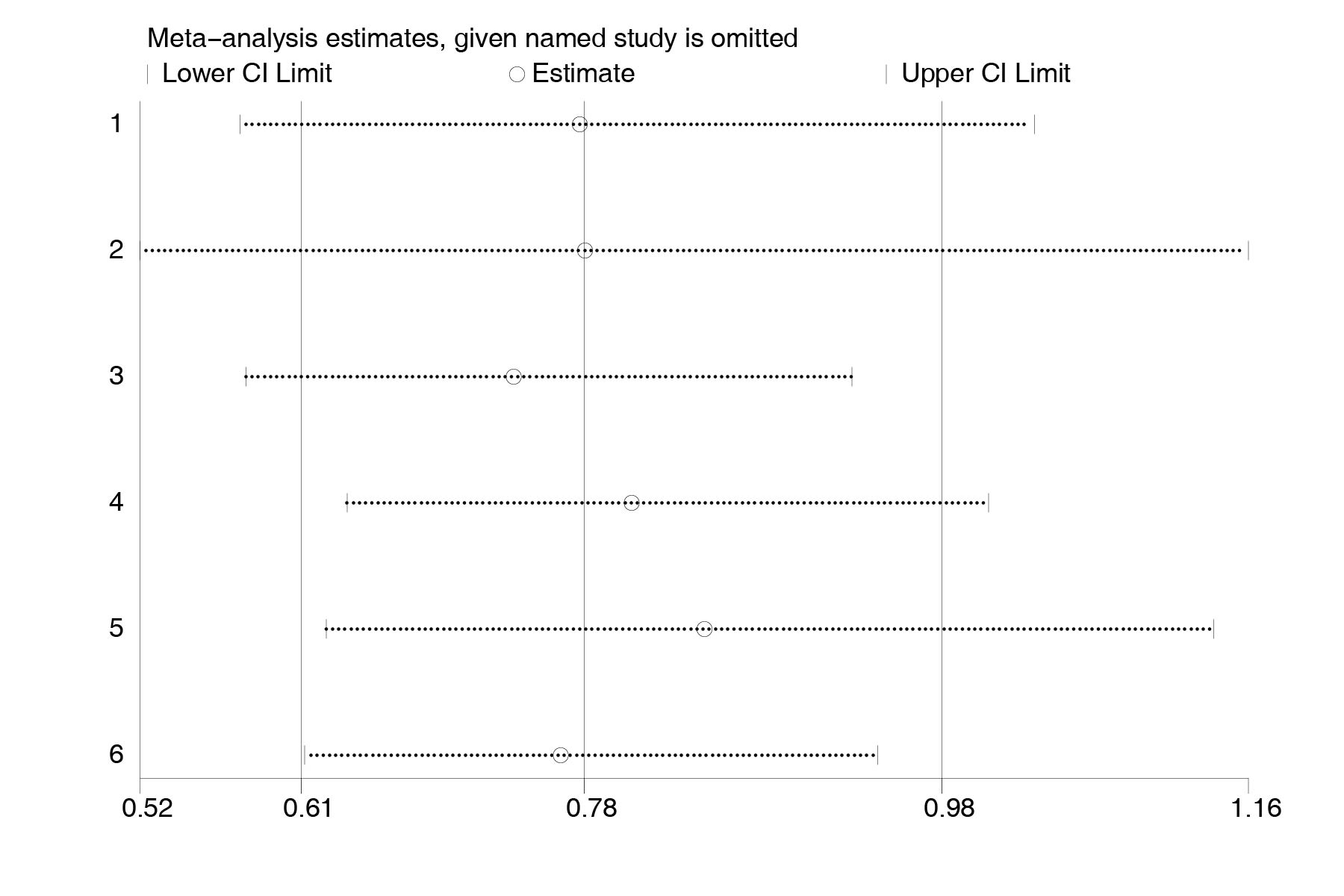

Supplement: Supplementary Figure 5 — Sensitivity analysis of SGLT2i versus Non-SGLT2i. [file Image_5.jpeg]

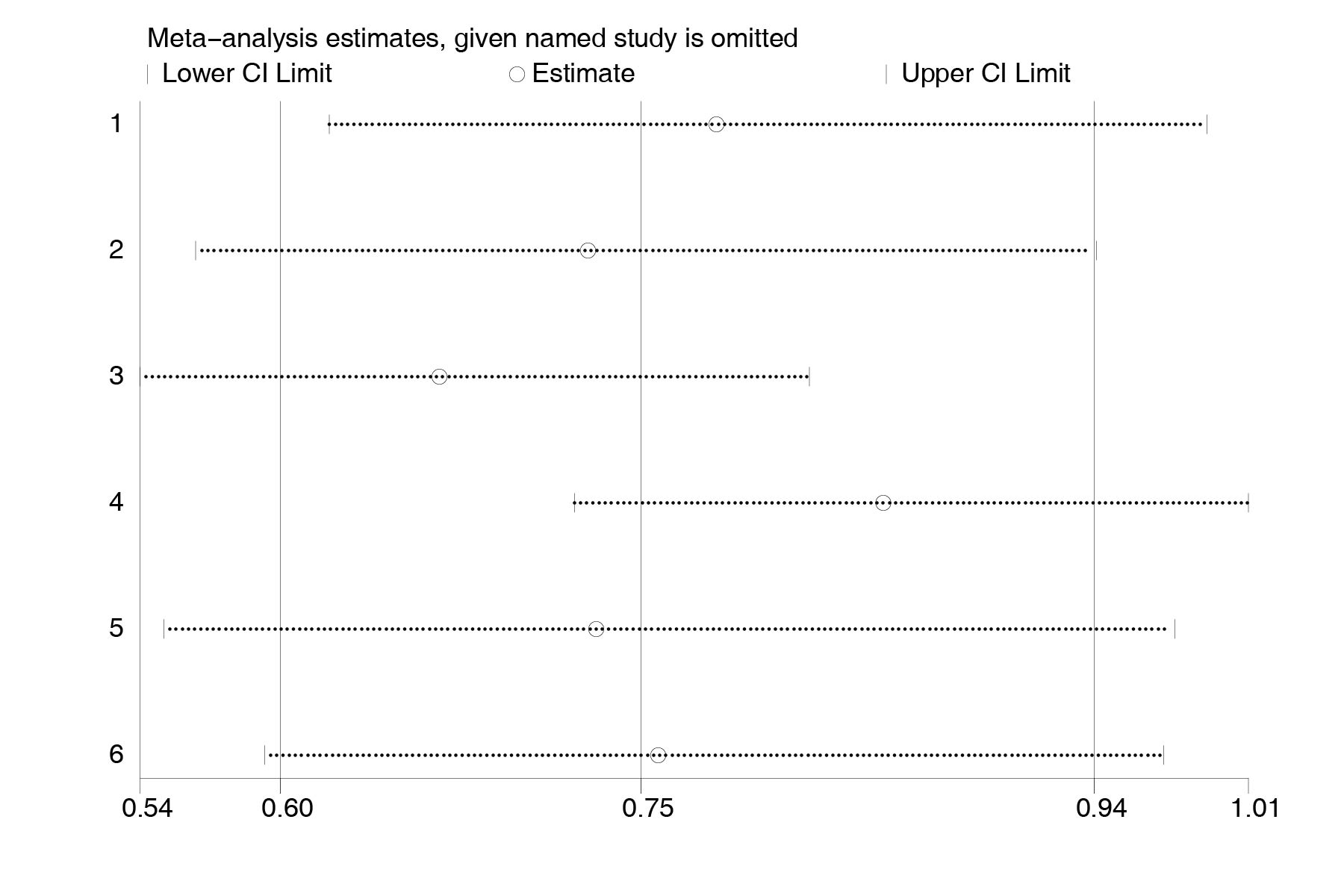

Supplement: Supplementary Figure 6 — Sensitivity analysis of GLP1RA versus Non-GLP1RA. [file Image_6.jpeg]
